# Evidence of new strains of *Wolbachia* symbiont colonising semiaquatic bugs (Hemiptera: Gerroidea) in mangrove environment of the Lesser Antilles

**DOI:** 10.1371/journal.pone.0273668

**Published:** 2022-08-30

**Authors:** Suzanne Conjard, Damien F. Meyer, Rosalie Aprelon, Nonito Pagès, Olivier Gros

**Affiliations:** 1 Institut de Systématique, Évolution, Biodiversité (ISYEB), Muséum national d’Histoire naturelle, CNRS, Sorbonne Université, EPHE, Université des Antilles Pointe-à-Pitre, Guadeloupe, France; 2 CIRAD, UMR ASTRE, Petit-Bourg, Guadeloupe, France; 3 ASTRE, Université Montpellier, CIRAD, INRA, Montpellier, France; Uppsala University: Uppsala Universitet, SWEDEN

## Abstract

*Wolbachia* Hertig, 1936 is an intracellular bacterial symbiont colonizing many arthropods. Of the studies done on the bacteria present in the superfamily Gerroidea Leach, 1815, no report of *Wolbachia* infection had yet been made. Thus, we checked the presence of *Wolbachia* in six Gerroidea species which colonize tropical aquatic environments by PCR using *wsp* primer set before sequencing and phylogenetic analyses. Insects were collected in the marine fringe of mangroves, in river estuaries, in swampy mangroves, and in ponds from Guadeloupe islands (Caribbean). Two new strains of *Wolbachia* were detected in these Gerroidea. They were named *w*Lfran and *w*Rmang. The *wsp* sequences suggest that the strains belong to the already described E supergroup or similar. *w*Lfran is present in *Limnogonus franciscanus* Stål, 1859 and *Rheumatobates trinitatis* (China, 1943) while *w*Rmang appears to be present exclusively in *R*. *mangrovensis* (China, 1943). Three other species were analysed, but did not appear to be infected: *Brachymetra albinerva* (Amyot & Serville, 1843), *Halobates micans* Eschscheltz, 1822, and *Microvelia pulchella* Westwood, 1834. The results presented here highlight for the first time the presence of new intracellular *Wolbachia* strains in Gerroidea colonising tropical aquatic environments like mangrove habitats from inlands to sea shore.

## Introduction

*Wolbachia pipientis* commonly known as *Wolbachia*, is an alpha-proteobacterium belonging to the Rickettsiales Gieszczykiewicz, 1939 order. It is mainly present intracellularly in the genital and somatic tissues of more than 60% of arthropods [1] and in nematodes [2]. In arthropods, the *Wolbachia* bacterium is mainly known for its ability to manipulate reproduction of the infected host, and can lead to production of infected females without males (inducing parthenogenesis), feminization of genetic males (inducing feminization), destruction of infected male offspring (killing males) and cytoplasmic incompatibility between infected males and uninfected (or infected with another strain) females [3].

To detect the presence of *Wolbachia*, a couple of methods are possible. The FISH (Fluorescence *In Situ* Hybridization) technic with the use of specific probes to visualize the bacteria within the insect tissues [4,5], or PCR (Polymerase Chain Reaction) targeting specific genes. The first detections of *Wolbachia* thanks to PCR were made using universal 16S rDNA primer sets and then using the *ftsZ* gene, which was considered more specific at the time [1,6]. Subsequently, PCR targeting the specific *wsp* gene (*Wolbachia* surface protein) which encodes a major protein of the cell surface layer became widespread [7]. Primers encoding *wsp* allow a preliminary identification of the *Wolbachia* strain. However, these last two decades the MLST (Multilocus Sequence Typing) method, which combines a set of genes, has become more popular [8]. This technique avoids the high recombination rates found with the *wsp* technique, which allows for a more reliable phylogeny [9]. More recently though the MLST and *wsp* typing system has been highly criticized, and whole genome sequencing and phylogenomic approaches seem to be best [10,11].

The phylogeny of *Wolbachia* is divided in various supergroups. Early phylogenetic analyses done almost 30 years ago determined the presence of two supergroups A and B, grouping together *Wolbachia* strains infecting different arthropods [1]. Then, *Wolbachia* strains phylogenetically related to supergroups C and D have been observed in filarial nematode hosts [12]. Today, the use of the MLST technique allows the identification of 19 supergroups up to the letter S [13].

Insects of the super-family Gerroidea are semi aquatic bugs belonging to the order Hemiptera. They live on the surface of the water, in rivers, estuaries or even in the sea. In tropical environments, they are also present in mangroves [14]. These semiaquatic insects move on the surface of the water thanks to a bunch of small bristles at the end of their legs [15]. They are sucking biting insects that feed opportunistically depending on the prey present on the surface of the water [15,16].

Apart from the wealth of publication on *Wolbachia* in many fields, there are only few studies on aquatic Hemiptera. According to research in Missouri, 52% of aquatic insects are infected with *Wolbachia*, compared to 60% in terrestrial insects [17]. Few recent studies demonstrated the presence of *Wolbachia* in semiaquatic bugs. In Cameroon, Esemu *et al*. (2019) detected *Wolbachia* in freshwater insects that represent a potential reservoir for the bacterium *Mycobacterium ulcerans* MacCallum, 1948 causing Buruli ulcer. This research focused on nine species of the Gerridae Leach, 1815 and Veliidae Amyot & Serville, 1843 families including the three genera *Limnogonus*, *Microvelia*, and *Rhagovelia* [18]. More recently, Castillo *et al*. [19] described the microbiome (including *Wolbachia*) associated with six species of gerrids belonging to the genera *Platygerris*, *Potamobates*, *Rheumatobates*, and *Telmatometra* from Panama [19].

Semi-aquatic bugs in the Lesser Antilles are not well known. A few studies confirmed their presence in these islands, the first focusing on Trinidad [20] and the second on Saint Martin [21]. For Guadeloupe, only two recent studies listing the insects of the island confirm the presence of six Gerroidea: *Brachymetra albinerva*, *Limnogonus franciscanus*, *Microvelia pulchella*, *Rhagovelia plumbea*, *Rheumatobates mangrovensis* and *R*. *trinitatis* [14,22]. Our study on *Wolbachia* in the Gerroidea of Guadeloupe focuses on six gerrids. *Brachymetra albinerva* lives in puddles between the roots of *Pterocarpus officinalis* in the swamp forest. *Limnogonus franciscanus* has the largest range, occurring on the terrestrial fringe of the mangrove, in the swamp forest and in the freshwater pools of “Grande-Terre” [14]. *Halobates micans* is exclusively marine, occurring in waves on the coast [23]. *Microvelia pulchella* is present in freshwater pools [22]. *Rheumatobates mangrovensis* is only present downstream of the “Grande-Rivière à Goyaves”. *Rheumatobates trinitatis* is also a marine species but prefers the mangrove waters more protected from wind and swell by mangrove trees, *Rhizophora mangle* Linnaeus, 1753 [14].

There is a great variability of *Wolbachia* strains in Arthropods [24]. Depending on geographical isolation or living environment, a host or strain of *Wolbachia* will change. For example, the fly *Bactrocera dorsalis* Hendel, 1912 in China is infected by four different strains throughout the country [25] or three species of the same fly genus can share one strain in South Asia [26]. Knowing this, it is interesting to demonstrate the presence of the bacterium in Gerroidea of Guadeloupe in order to have a first idea of the prevalence of the bacterium on the island. In order to detect the presence of *Wolbachia* symbionts in Gerroidea, we solely use PCR with primers specific for the *wsp* gene.

## Materials and methods

### Sampling

**Fig 1:**
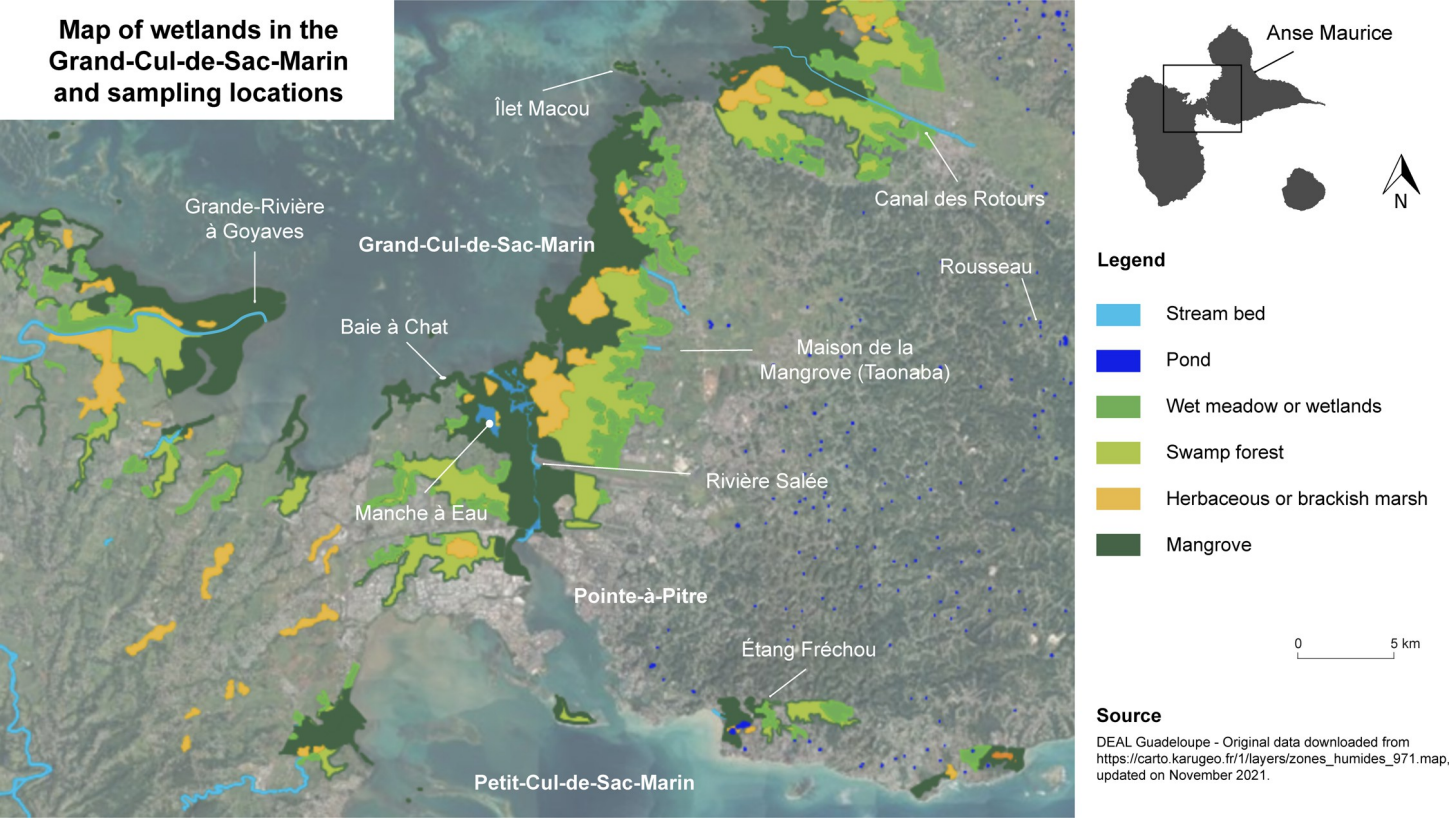
Map of wetlands in Guadeloupe and associated sampling sites. https://carto.karugeo.fr/1/layers/zones_humides_971.map.

Sampling took place between 2019 and 2021, in the mangrove of the “Grand-Cul-de-Sac-Marin”, in the swampy forest of the back mangrove at the “Maison de la Mangrove”, in a pond of Grande-Terre “Étang Fréchou”, and on the shoreline coast of the Atlantic side of Grande-Terre “Anse-Maurice” (Fig 1). The samples were taken by boat in the marine mangrove and by feet for the other sites, using a 1 mm-mesh size nylon net. Apart from the collection sites “Anse-Maurice” and “Étang Fréchou”, all other insect collection sites are located in the marine area adjacent to the “Parc National de la Guadeloupe” or in the case of the “Grande-Rivière à Goyaves” estuary in the heart of the “Parc National de la Guadeloupe” (“permit n°2019–15”).

A total of 60 individuals of Gerroidea were collected and analyzed in different sampling sites according to their habitats (Fig 1 and Table 1). Where possible, for each collection, males, females and juveniles were analyzed.

**Table 1:** Number of samples per species and per sampling site.

Species	Sampling site (Fig 1)	Number of samplings
***Brachymetra albinerva***	Maison de la Mangrove	6
***Halobates micans***	Anse-Maurice	6
***Limnogonus franciscanus***	Canal des Rotours	10
	Rousseau	2
	Maison de la Mangrove	8
***Microvelia pulchella***	Étang Fréchou	5
***Rheumatobates mangrovensis***	Estuary of “Grande-Rivière à Goyaves”	8
***R*. *trinitatis***	Baie à Chat	3
	Canal des Rotours	2
	Ilet Macou	1
	Manche à Eau	8
	Rivière Salée	1

### Identification of insects, PCR amplification, and sequencing

The collected Gerroidea were individually identified using integrative taxonomy with two methods. Taxonomic determination keys [15,23,27–30] and PCR amplification followed by sequencing of the PCR products of the gene encoding COI (Cytochrome Oxidase subunit I) using the primers set *LCO1490F* and *HCO2198R* [31]. DNA was extracted using the DNeasy Blood & Tissue Kit following the manufacturer’s protocol. PCR was performed under the following conditions: initial denaturation at 94°C for 4 minutes, 30 denaturation cycles (94°C, 60 seconds), annealing (55°C, 45 seconds), extension (72°C, 45 seconds) and a final extension at 72°C for 1 minute. The expected size of the PCR products was checked after 2% agarose gel electrophoresis before to be directly sequenced by Eurofins (http://www.eurofinsgenomics.eu).

When identified, insects were selected in a representative manner according to species and habitat location. The primer set used for PCR was that corresponding to the *Wolbachia wsp*-specific surface protein (*wsp81F* and *wsp691R*) developped by Duron et al. (2008) [32]. PCR was performed under the following conditions: initial denaturation at 94°C for 3 minutes, 40 denaturation cycles (94°C, 60 seconds), annealing (54°C, 60 seconds), extension (72°C, 1 minute) and a final extension at 72°C for 7 minutes. The expected size of the PCR product is 610 bp. PCR products were verified after electrophoresis on 2% agarose gel electrophoresis. The positive control used is DNA from *Rheumatobates mangrovensis* that has been shown to be positive. The negative control is ultra-pure water. The PCR products were then directly sequenced (from the both primers used) by Eurofins (http://www.eurofinsgenomics.eu).

### Phylogenetic analysis

**Fig 2:**
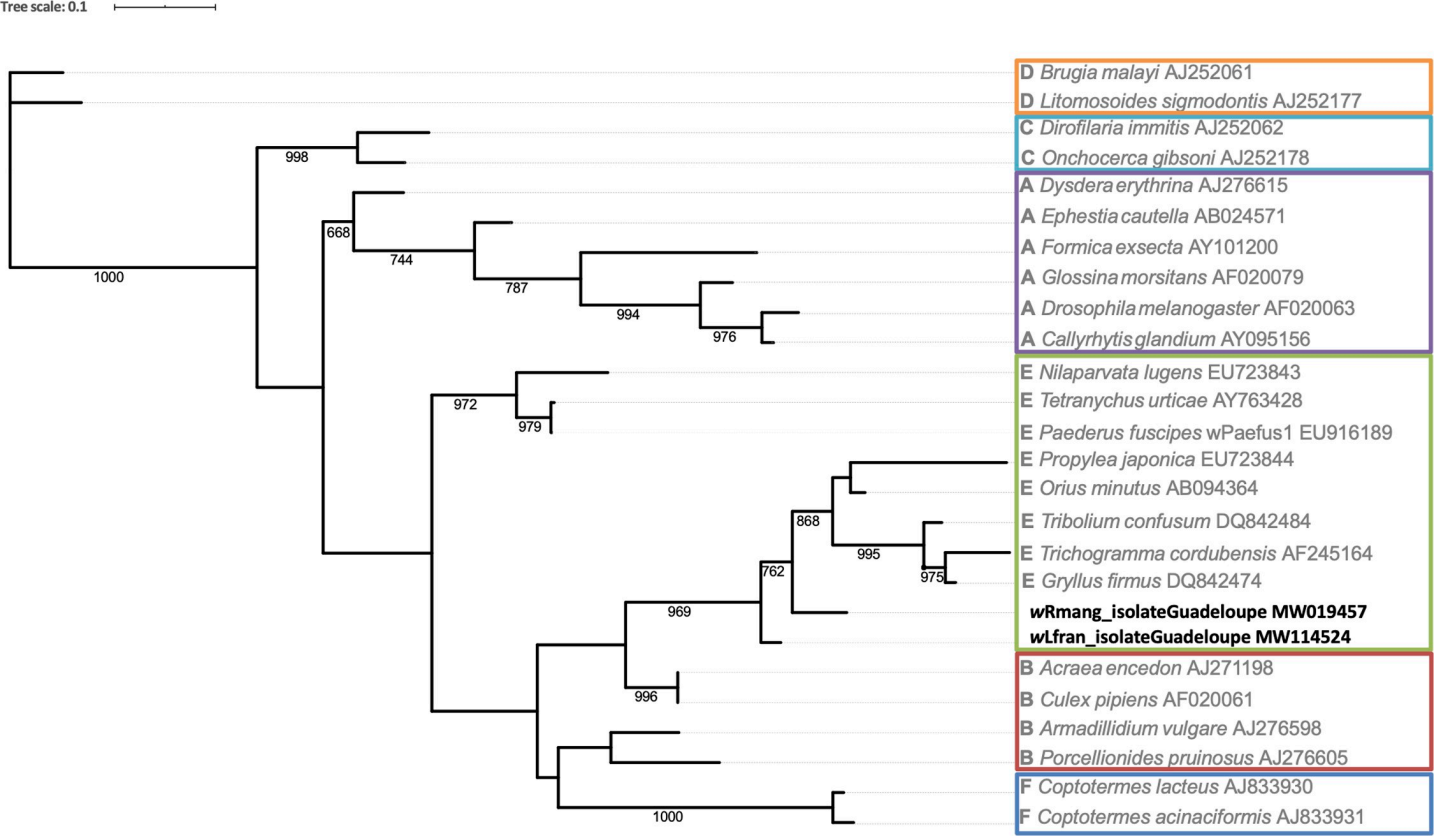
Phylogenetic tree of *Wolbachia* detected in Gerroidea and other insects based on their *wsp* sequences. Tree was inferred from maximum likelihood method using PhyML program and drawn using iTOL program. Branches are labelled as Supergroup Letter and specie names followed by GenBank accession numbers. Bootstrap values (1,000 replicates) are indicated, except for branches with a value less than 0.5.

*Wolbachia wsp* sequences from gerrids used for phylogenetic analysis were downloaded from NCBI (Table 3 and Fig 2) in addition to the sequences obtained in this study (MW019457 and MW114524). They were all analyzed and aligned with Geneious (Geneious version R10, https://www.geneious.com) and MAFFT [33]. The phylogenetic tree was constructed using PhyML software [34] with a maximum likelihood approach. The bootstrap analysis was performed with 1000 replicates, and the bootstrap values were calculated using a 50% majority rule.

## Results

### *Wolbachia* infection in Gerroidea of Guadeloupe

*Wolbachia* were detected in three of the six species tested: *L*. *franciscanus*, *R*. *mangrovensis* and *R*. *trinitatis*. *Wolbachia* was detected in all *R*. *mangrovensis* and *L*. *fransciscanus* individuals while occasionally in *R*. *trinitatis* and not detected in *B*. *albinerva*, *H*. *micans* and *M*. *pulchella* tested individuals (Tables 2 and 3).

**Table 2:** Table showing the prevalence of *Wolbachia* in Gerroidea samples collected at different collection sites.

Species	Number of *Wolbachia* negative individuals	Number of *Wolbachia* positive individuals	Sequenced samples
***Brachymetra albinerva***	6	0	0
***Halobates micans***	6	0	0
***Limnogonus franciscanus***	0	14	6
***Microvelia pulchella***	6	0	0
***Rheumatobates mangrovensis***	0	8	5
***R*. *trinitatis***	6	9	3

**Table 3:** Survey of the sequences obtained and their existing matches in BLAST.

Results of the study				BLAST match		
Species	Sample number	Sampling site (Fig 1)	Number of nucleotides obtained	Species name	Percent identity	Accession number
***Limnogonus franciscanus***	#1	Canal des Rotours	467	*Dichocrocis punctiferalis*	96.78%	GU166597.1
***Limnogonus franciscanus***	#2	Maison de la Mangrove	434	*Pteromalus puparum*	97.51%	DQ493917.1
***Limnogonus franciscanus***	#3	Maison de la Mangrove	439	*Dichocrocis punctiferalis*	97.27%	GU166597.1
***Limnogonus franciscanus***	#4	Maison de la Mangrove	490	*Pteromalus puparum*	97.53%	DQ493917.1
***Limnogonus franciscanus***	#5	Maison de la Mangrove	512	*Pteromalus puparum*	97.45%	DQ493917.1
				*Ceroptres cerri*	97.25%	AY095157.1
				*Dichocrocis punctiferalis*	96.63%	GU166597.1
***Limnogonus franciscanus***	#6	Rousseau	517	*Dichocrocis punctiferalis*	96.90%	GU166597.1
***Rheumatobates trinitatis***	#1	Canal des Rotours	489	*Pteromalus puparum*	97.73%	DQ493917.1
				*Ceroptres cerri*	97.53%	AY095157.1
				*Dichocrocis punctiferalis*	96.93%	GU166597.1
***Rheumatobates trinitatis***	#2	Îlet Macou	508	*Pteromalus puparum*	97.43%	DQ493917.1
				*Ceroptres cerri*	97.23%	AY095157.1
				*Dichocrocis punctiferalis*	96.65%	GU166597.1
***Rheumatobates trinitatis***	#3	Manche à Eau	512	*Pteromalus puparum*	97.25%	DQ493917.1
				*Ceroptres cerri*	97.05%	AY095157.1
				*Dichocrocis punctiferalis*	96.48%	GU166597.1
***Rheumatobates mangrovensis***	#1	Estuary of “Grande-Rivière à Goyaves”	358	*Delphacodes kuscheli*	93.58%	KM386825.1
***Rheumatobates mangrovensis***	#2	Estuary of “Grande-Rivière à Goyaves”	518	*Macrolophus pygmaeus*	94.56%	FJ374283.1
***Rheumatobates mangrovensis***	#3	Estuary of “Grande-Rivière à Goyaves”	518	*Stephanitis pyrioides*	94.56%	AB109622.1
***Rheumatobates mangrovensis***	#4	Estuary of “Grande-Rivière à Goyaves”	571	*Delphacodes kuscheli*	93.33%	KM386825.1
***Rheumatobates mangrovensis***	#5	Estuary of “Grande-Rivière à Goyaves”	595	*Macrolophus pygmaeus*	93.21%	FJ374283.1

### Sequence and phylogenetic analyses

We amplified and partially sequenced the *wsp* gene (Table 3). From these sequences, two major *Wolbachia* strains were detected in Gerroidea from Guadeloupe and registered in the NCBI database: *w*Rmang_isolateGuadeloupe (MW019457) and *w*Lfran_isolateGuadeloupe (MW114524).

The *w*Rmang strain was detected exclusively in *Rheumatobates mangrovensis* species, from the unique place where they can be found at the mouth of the river “Grande-Rivière à Goyaves” (Fig 1 and Table 1). The *w*Lfran strain is the most prevalent strain and is detected in *Limnogonus franciscanus* and *R*. *trinitatis*. The two species do not share the same habitat. However, they can be found occasionally at sites such as the “Canal des Rotours” (Fig 1), a watercourse that crosses many environments, wet meadows, swamp forest and leads to mangroves. *Limnogonus franciscanus* is a freshwater species that prefers habitats like puddles or even private fountains in gardens like small or a natural pond at the “Maison de la Mangrove”. *Rheumatobates trinitatis* is a marine species that will prefer sheltered mangrove environments such as at the “Manche à Eau” or in the “Baie à Chat” (Fig 1). The two strains have 93% homogeneity between them.

The phylogenetic tree, based on *wsp* gene sequences, shows the distinct presence of *w*Lfran and *w*Rmang strains within the cluster of E-supergroup sequences (Fig 2).

## Discussion

In the present study, six species of semi-aquatic bugs were analyzed by PCR and sequenced to detect and identify *Wolbachia* among Gerroidea from Guadeloupe island (Caribbean, Lesser Antilles). Thus, three Gerroidea species are infected by two new strains (*w*Lfran and *w*Rmang) belonging to E supergroup. This study provides first insight for the prevalence of *Wolbachia* in Gerroidea in Guadeloupe.

*Wolbachia* strains have been reported on arthropod pests such as scorpion [35], flies [36], terrestrial isopods [4], and spiders [37]. However, the majority of these studies have focused on insects with an impact on health or agriculture. *Wolbachia* has been detected in a wide diversity of mosquito species, some of them vectors of medically important arboviruses [38]. The *Wolbachia* bacterium has been proposed as a regulatory tool for Zika and/or dengue bearing mosquitoes [39–41]. Studies on the occurrence of *Wolbachia* in insect pests of crops mainly concern Diptera [25,26], Acari [24,42] and phytophagous Hemiptera [43–48]. Of all the studies carried out, very few have been conducted on aquatic insects and even fewer on marine insects. However, two recent studies conducted in Cameroon [18] and Panama [19] looking for the bacterial community in the Gerridae and Veliidae found *Wolbachia* in these taxa.

In Guadeloupe, *Wolbachia* has already been studied in some mosquito species: *Aedes aegypti* Linnaeus, 1762, *A*. *taeniorhynchus* (Wiedemann, 1821) and *Culex quinquefasciatus* Say, 1823 mosquitoes [49,50], *Deinocerites* sp. Theobald, 1901, *Mansonia* sp. Blanchard, 1901, *Ochlerotatus* sp. Reinert, 2000 and *Uranotaenia* sp. Lynch Arribálzaga, 1891 [51]. While mosquitoes of the genus *Aedes* are usually free of *Wolbachia*, this is not the case for *Cx*. *quinquefasciatus*, which has a variable infection rate of between 79 to 96% [49]. Mosquitoes, although temporarily aquatic insects, do not appear to share common *Wolbachia* strains with Gerroidea based on results of the present study.

The low number of Gerroidea samples collected during this study does not allow statistical treatment of data. However, these preliminary results suggest a high presence of *Wolbachia* in certain species such as *L*. *franciscanus*, *R*. *mangrovensis* and *R*. *trinitatis* and an absence, or very low presence, in *B*. *albinerva*, *H*. *micans* and *M*. *pulchella*. Given that the sampling effort is really different between Gerroidea and Guadeloupean mosquitoes, a pattern of infection appears to be emerging. Like the *Aedes* mosquitoes from Guadeloupe, *B*. *albinerva* and *M*. *pulchella*, while sharing the same environment as the other Gerroidea tested, does not appear to be infected. Thus, the presence of *Wolbachia* seems to be related to the insect species rather than to the environment occupied. *Wolbachia* infection is known to be variable within a population of the same genus but also within a same species depending on its geography [25,26,44]. Depending on its geographical origin, the frequency of *Wolbachia* infection can be very variable, ranging from 4–100% in one sampling area to 37–100% in another [44]. Statistically, the intraspecific infection rate can be very high or very low and there is no average infection rate [52]. In some genera, depending on the species, the average infection rate varies from quite low at less than 3% [24] to very high with an infection rate close to 100% [47]. In view of the different forms of infection, it is therefore normal to observe variations in infection among the six species studied in Guadeloupe.

Currently, taking into account all invertebrates and all analytical methods, there are nineteen *Wolbachia* supergroups named from A to S (G is not included because it is a grouping of A and B supergroups) [13,24,26,42]. In our study, *Wolbachia* symbionts infecting mangrove Gerroidea belong to E supergroup according to *wsp* phylogenetic analysis (Fig 2).

The *Wolbachia* strain colonizing the pirate bug *Orius minutus* (Linnaeus, 1758) is also part of E supergroup. This Anthochorid belongs to the order Hemiptera, but remains genetically distant from the ones analyzed in this study as is not part of the superfamily Gerroidea. In contrast, *Gerris* sp. Fabricius, 1794 from temperate habitats has been found to be positive for *Wolbachia* strains of the B supergroup in England [53] but negative in France [32]. Overall, few studies have been conducted and updated on *Wolbachia* E supergroup, but it seems that several hosts are possible, including insects, spiders [37] and springtails [54]. Therefore, there is currently little data regarding the occurrence of *Wolbachia* in the superfamily Gerroidea and the prevalence of E supergroup is not yet known. Typing of strains into supergroups was mainly based on phylogenetic inference of the *Wolbachia* surface protein (*wsp*) which is easy to implement and reproducible. However, the small size of this gene and its high recombination rate make it unreliable for precise phylogenetic analyses [8]. Therefore, a full characterization of the MultiLocus Sequence Typing (MLST) is necessary for a correct typing of the strain [8]. MLST analysis is based on the study of several genes. This technique allows refining the analysis by increasing the number of supergroups detected, 16 different supergroups have been described so far, without changing the overall phylogeny [26,55]. However, this technique is nowadays discussed because the markers used are not discriminating enough and are not reliable enough at fine-scale [10].

It is now possible to state that Gerroidea from Guadeloupe mangroves are infected by different *Wolbachia* strains. However, as the use of *wsp* gene sequences is not sufficiently discriminating, it would be interesting to strengthen the research with other methods such as the analysis of the whole genome sequence of the strains which remains the most reliable method [10,56]. This later technique would allow us to consolidate the results and possibly confirm the presence of these strains in E supergroup.

Knowledge of aquatic insects in marine, brackish and lacustrine environments in the tropics is poor. In Guadeloupe, other *Wolbachia* strains have been observed in spiders and ants from swamp and mangrove forests [57]. Increasing our knowledge on the diversity of *Wolbachia* strains colonizing various insects from similar environments through the island would allow us to demonstrate the strategy used by these symbiotic *Wolbachia* strains in such tropical Hemiptera to be transmitted to the new host generations.

## Nucleotide sequence accession number

*Wolbachia wsp* gene partial sequences obtained in this study were deposited in the Genebank database under accession numbers MW019457 and MW114524.
